# Three Trends Shaping the Politics of Aging in America

**DOI:** 10.1093/geroni/igaa057.2367

**Published:** 2020-12-16

**Authors:** Nora Super

**Affiliations:** Milken Institute, Washington, District of Columbia, United States

## Abstract

The demographic bulge created by the baby boom generation has shaped American politics since they came of age in the 1960s. Over the next decade, aging issues will become more relevant as the oldest boomers reach 84 and the youngest boomers will be eligible for Medicare. This paper highlights three converging trends that will shape United States politics; including increased spending on “entitlement” programs like Social Security and Medicare, growing mismatch in caregiving need and supply, and the heightened concentration of older adults in certain geographic areas. The next decade will see not only extraordinary demographic change but also unprecedented advances in technology and medicine, and cultural and societal shifts that were once unimaginable.

